# Pregnancy Outcomes of Conservative Management in Preeclampsia with Severe Features

**DOI:** 10.3390/jcm12196360

**Published:** 2023-10-04

**Authors:** Anuchit Inta, Theera Tongsong, Kasemsri Srisupundit

**Affiliations:** Department of Obstetrics and Gynecology, Faculty of Medicine, Chiang Mai University, Chiang Mai 50200, Thailand

**Keywords:** expectant management, preeclampsia, pregnancy outcome, severe feature

## Abstract

***Objective:*** To study the pregnancy outcomes of conservative treatment for preeclampsia with severe features. ***Methods:*** A retrospective study was conducted on pregnancies with preeclampsia with severe features at gestational age 23–34 weeks and that received conservative management at Chiang Mai University Hospital between January 2014 and August 2020. The women were divided into two groups: (1) pregnancy prolongation of at least 48 h and (2) pregnancy prolongation of less than 48 h. ***Results:*** Of the 100 recruited pregnancies, the median gestational age was 29 weeks (range 23–34). Of these, 65 cases (65%) had pregnancy prolongation of at least 48 h, and 35 cases (35%) had prolongation of less than 48 h. The median pregnancy prolongation was 2.9 days (range 4 h–27.7 days). Eighty-seven (88%) pregnant women experienced no complications. Multivariate analysis shows that high urine protein/creatinine ratio (UPCI) at admission was significantly associated with pregnancy prolongation of less than 48 h with an odds ratio for prolongation for at least 48 h of 0.86 (95% CI 0.75–0.99: *p*-value 0.04). Kaplan–Meier analysis shows that the mean time of prolongation was 3.6 days vs. 6.7 days, and median time of prolongation was 2.1 days vs. 4.4 days in the group of high and low UPCI (using cut-off 1.0), respectively. The number of prolonged days was significantly lower in the high UPCI group than in the low UPCI group (log-rank test, *p* = 0.01). The maternal and fetal outcomes between the two groups were not significantly different. The cesarean section rate was also comparable. The mean birth weight and gestational age at delivery were not significantly different, though they had a higher trend in the group of successful conservative management. Conclusion: The rate of pregnancy prolongation of at least 48 h with conservative management was 65%, with a median prolongation time of 2.9 days. A new insight gained from this study is that high UPCI at admission is an independent factor for prolongation of less than 48 h with conservative treatment. Nevertheless, the maternal and fetal outcomes between the two groups were not significantly different. Therefore, the benefit and risk of expectant management in actual practice of service settings in terms of maternal and fetal morbidity is still unclear.

## 1. Introduction

Preeclampsia is one of the leading causes of maternal death. Moreover, preterm preeclampsia is also the common cause of neonatal death secondary to prematurity. Therefore, there is an option of conservative (or expectant) management in pregnancy with preterm preeclampsia with severe features, primarily aimed at reducing neonatal morbidity and mortality from induced preterm delivery. Expectant management mainly consists of a delay in labor induction, administration of magnesium sulfate for convulsion prevention, antihypertensive drugs to control blood pressure, and corticosteroids to promote fetal lung maturity. There have been many studies on the role of expectant management in cases of preterm preeclampsia with severe features, which showed that this not only reduced neonatal morbidities but was also safe for mothers [1]. Currently, expectant management has become more commonly practiced, with the caveat that women with HELLP syndrome or growth-restricted fetuses were usually excluded. Nevertheless, based on a recent meta-analysis study, although an expectant approach to the management of women with severe early-onset pre-eclampsia may be associated with decreased neonatal morbidity, further studies are needed to establish whether this approach is safe for the mother [2]. Furthermore, most studies with favorable outcomes of the expectant approach were conducted in tertiary centers with available facilities and care providers in Western countries. However, studies on expectant management in low-to-middle resource countries are very limited [3,4,5]. According to the American College of Obstetricians and Gynecologists (ACOG) guidelines [6], expectant management of preeclampsia with severe features prior to 34 weeks of pregnancy must be based on strict selection criteria and should be performed in a setting with available resources for maternal and neonatal care. Nevertheless, expectant management is practiced in many countries with various availabilities of medical resources, and its outcomes have never been thoroughly evaluated. This study is based on actual practice, further studies of which are still needed in the literature. Though there have been several studies published upon the subject, the recent meta-analyses suggest that the benefit of expectant management is still unclear, and more studies are needed. Not only are RCT or prospective studies in ideal conditions of research settings needed but also studies based on service settings or real practice after implementation of the guidelines. Therefore, this study aimed to explore pregnancy outcomes, including maternal and neonatal outcomes, of expectant management of preeclampsia with severe features in actual practice.

## 2. Materials and Methods

A retrospective study was conducted using the database of pregnancies with preeclampsia with severe features at gestational ages between 23 and 34 weeks that received expectant management at Chiang Mai University hospital from 1 January 2014 to 31 August 2020. This study was conducted with ethical approval from the Institutional Review Board (Research Ethics Committee 4; Faculty of Medicine, Chiang Mai University (Research ID: OBG 2564-08255). The maternal–fetal medicine database was accessed, and all consecutive records of patients diagnosed with preeclampsia with severe features were screened and retrieved. In addition, the medical records were comprehensively reviewed and validated for inclusion. The definition of severe features used in this study included any of the following clinical criteria of preeclampsia: (1) systolic blood pressure of at least 160 mm Hg or diastolic blood pressure of at least 110 mm Hg on two occasions at least 4 h apart (unless antihypertensive medication was administered before this time); (2) thrombocytopenia (platelet count of less than 100,000 per microliter); (3) impairment of liver function associated with preeclampsia and as documented by abnormally increased blood levels of aspartate aminotransferase (AST) or alanine aminotransferase (ALT) (to more than twice the upper limit normal levels); (4) severe persistent epigastric or right upper quadrant pain unresponsive to medications; (5) renal impairment defined as serum creatinine concentration more than 1.1 mg/dL or a doubling of the serum creatinine concentration in the absence of other renal disease; (6) pulmonary edema; (7) new-onset headache with no response to painkillers and without underlying disease; (8) visual disturbances or neurological deficit; (9) eclampsia. In our practice, expectant management of preeclampsia with severe features was contraindicated in cases with the following conditions: (a) persistent headaches, unresponsive to analgesics; (b) visual disturbances, alteration of consciousness, or motor dysfunction; (c) uncontrolled severe-range blood pressures (persistent systolic blood pressure of 160 or more or diastolic blood pressure of 110 mm Hg or more), unresponsive to antihypertensive therapy; (d) persistent epigastric pain or right upper quadrant pain unresponsive to medication; (e) stroke; (f) myocardial infarction; (g) HELLP syndrome; (h) new-onset or worsening renal impairment (serum creatinine greater than 1.1 mg/dL or twice baseline); (i) pulmonary edema; (j) eclampsia; (k) suspected placental abruption; (l) non-reassuring fetal well-being, such as persistent reversed end-diastolic flow in the umbilical artery, or fetal death. 

The inclusion criteria for this study included pregnant women who were diagnosed with preeclampsia with severe features or chronic hypertension with superimposed preeclampsia with severe features between 23 and 34 weeks of gestation and received expectant management on admission. Expectant management in our practice was summarized as follows: (1) initial intensive care and supportive care (such as fluid resuscitation) to stabilize the patient; (2) corticosteroid administration (48 h course) for fetal lung maturation; (3) magnesium sulfate for convulsion prevention as a standard intravenous regimen for 48 h; (4) treatment of hypertension to keep blood pressure under 160/90 mmHg using antihypertensive medications: labetalol, hydralazine, or nifedipine; (5) intake output monitoring; (6) daily assessment of fetal well-being, either non-stress test or biophysical profile; (7) daily maternal assessment such as vital signs, signs of severe features, or magnesium sulfate intoxication; (8) serial laboratory evaluation for HELLP syndrome and renal function; (9) delivery in cases of prolongation of pregnancy until gestational age of 34 weeks, preterm rupture of membranes after complete course of corticosteroids, or development of any contraindications mentioned above. 

The exclusion criterion was incomplete data, especially data on pregnancy outcomes. The women were then divided into two groups: (1) pregnancy prolongation of at least 48 h and (2) pregnancy prolongation of less than 48 h. The duration of pregnancy prolongation was calculated from the beginning of expectant management until delivery. Note that all patients in this study received magnesium sulfate for convulsion prophylaxis and antihypertensive medications to control blood pressure. Baseline characteristics of the study population, including blood pressure, urine protein/creatinine ratio (UPCI), and maternal and neonatal complications, were reviewed and recorded. The primary outcome was the percentage of pregnancy prolongation for at least 48 h with expectant management, while the secondary outcomes were maternal and neonatal complications.

***Statistical analysis:*** The analysis was performed using the Statistical Package for the Social Sciences (SPSS) software version 26.0 (IBM Corp. Released 2019. IBM SPSS Statistics for Windows, Version 26.0 IBM Corp: Armonk, NY, USA) using descriptive statistics, the Mann–Whitney U test, the chi-square test, univariate and multivariate analysis, and the Kaplan–Meier test, as appropriate. Statistical significance was defined as a *p*-value < 0.05. Based on a previous study by Garcia et al. [7], using composite morbidities of 55.6% as a primary outcome, this study needed a sample size of at least 95 cases to gain a power of 80% at a 95% confidence interval.

## 3. Results

Of the 100 pregnancies meeting the inclusion criteria, the median gestational age was 29 weeks (range 23–34). The median pregnancy prolongation was 2.9 days (range 4 h–27.7 days). Eighty-seven (88%) pregnant women experienced no complications. Of the 100 recruited pregnancies, 65 (65%) and 35 (35%) had pregnancy prolongation ≥ 48 h and prolongation < 48 h, respectively. The baseline demographic data of the group of prolongation of less than 48 h and that of at least 48 h are shown in Table 1. 

Chronic hypertension was the most common underlying disease, accounting for 20% of all cases, and its prevalence was not significantly different between the two groups. Regarding the pregnancy outcomes, the incidence of maternal and neonatal complications was not significantly different between the two groups as indicated in Table 2. Maternal obstetric complications included postpartum hemorrhage, pulmonary edema, and HELLP syndrome, whereas neonatal complications included respiratory distress syndrome and small size for gestational age. The most common neonatal complications were associated with prematurity, especially respiratory distress syndrome, which were found in approximately 45% of cases, comparable in both groups. The mean gestational age at delivery was not significantly different. However, note that gestational age in the successful group tended to be lower at the time of baseline but tended to be higher at delivery. Also, the mean birth weight was not significantly different, though there was a higher trend in the group of successful conservative management (1092 + 392 vs. 1234 + 482; p-value: 0.152). Concerning route of delivery, the rate of cesarean section was as high as 77%, comparable between the two groups (30 [85.7%] vs. 47 [72.3%]; *p*-value: 0.312). Notably, there was no maternal death in both groups, whereas early perinatal death (within 7 days of neonatal life) was documented in one case in each group.

Interestingly, though most of the baseline characteristics of the two groups were not significantly different, the UPCI on admission in the group of prolongation < 48 h was significantly higher than that in the group of prolongation ≥ 48 h (*p* = 0.006), as shown in Table 1. 

Univariate and multivariate analyses were performed to identify risk factors for pregnancy prolongation ≥ 48 h with expectant management (Table 3).

**Figure 1:**
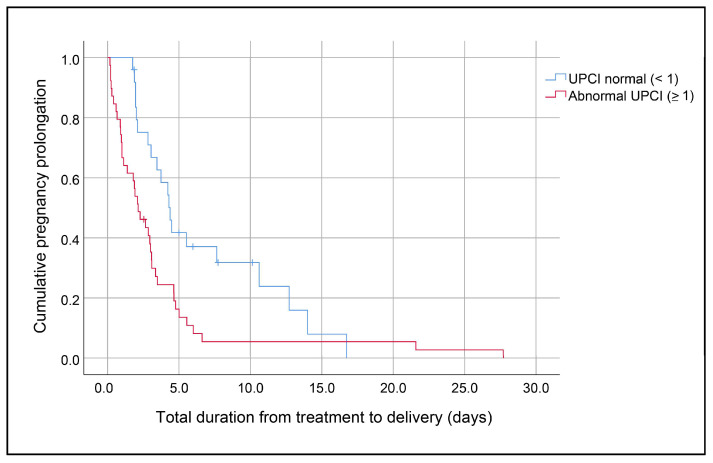
Pregnancy prolongation days between group of high and low UPCI (log rank test; *p*-value 0.01).

Multivariate analysis shows that high UPCI on admission was significantly associated with pregnancy prolongation < 48 h with an odds ratio for ≥ 48 h of 0.86 (95% CI 0.75-0.99: *p*-value 0.04). Kaplan–Meier analysis shows that the mean time of prolongation was 3.6 days vs. 6.7 days, and median time of prolongation was 2.1 days vs. 4.4 days in the group of high and low UPCI (using cut-off 1.0), respectively. The number of prolonged days was significantly lower in the high UPCI group (log-rank test; *p* = 0.01), as shown in Figure 1. The number of prolongation days was not significantly correlated with the gestational age of diagnosis (Pearson correlation; *p*-value: 0.139).

## 4. Discussion

Currently, expectant management in pregnant women with preterm preeclampsia with severe features is a treatment option to reduce neonatal morbidity from preterm birth, especially in cases with gestational age less than 34 weeks [6]. However, expectant management, which mainly focuses on delaying labor induction as much as possible, may cause maternal complications and require close monitoring from healthcare personnel. In many developing countries or geographical areas with low resources, expectant management of preeclampsia with severe features is very challenging. Although it has become a standard treatment and is well accepted in developed countries, it has never been proven safe or reproducible in the third world or in a low-resource setting [4]. It must be emphasized that in the standard guidelines, mothers undergoing expectant treatment must be taken care of by MFM specialists [6]. Although we have adopted this standard of care for more than 10 years in our hospital, the outcomes have not been thoroughly evaluated. Our study indicates that expectant treatment is relatively safe. The fetuses benefited from corticosteroid administration for lung maturity in most cases, although an additional risk to the mothers might exist, but this risk minimal and could be prevented with high precautions. Accordingly, the important findings of this study indicate that guidelines for expectant management may be adopted in low-to-middle resource countries. However, our hospital is defined as a tertiary care center in our country; therefore, the results of this study may not represent the outcomes of treatment in truly low-resource settings.

In the present study, the median duration of pregnancy prolongation was only 2.9 days, which is different from previous studies. For example, in a classic study by Sibai et al. [1], pregnancy was prolonged for a mean of 15.4 days in the expectant management group. Therefore, each country or healthcare setting should have its own study about the outcome of expectant management to evaluate risk–benefit thoroughly and make decisions regarding treatment options based on their own data. Most indications for discontinuing expectant management in our study were spontaneous labor, uncontrolled hypertension, obstetric complications, such as placental abruption, and non-reassuring fetal heart rate. Even though the median pregnancy prolongation in this study was only 2.9 days, this period was long enough for the administration of corticosteroids to promote fetal lung maturity. Therefore, this study suggests that expectant management of preterm uncomplicated preeclampsia with severe features may be offered to prolong gestational age by at least 48 h for corticosteroid administration. Prolongation for more than 48 h should be considered individually. Risk–benefit after this should be strongly considered on a case-by-case basis, depending on resources, gestational age, and difficulty in hypertension control. Based on this study and previous studies [7,8,9,10,11,12], expectant management is beneficial to perinatal outcomes. However, these data were insufficient to establish conclusions regarding maternal health. This is because maternal complications, such as placental abruption, HELLP syndrome, pulmonary edema, renal failure, and eclampsia, are relatively rare but very important and must be taken into consideration to weigh the benefits of expectant management, especially in low-resource settings. In addition, it should be noted that the duration of prolongation in actual practice, as seen in our study, may differ from that reported in research settings. 

In the literature review, in most prospective controlled studies, expectant management can result in prolongation of pregnancy in approximately 7–15 days [1,4,5,7,13,14], but only one-third of patients remained pregnant beyond seven days [14]. Nevertheless, the duration of prolongation seems to be much shorter in observational studies based on actual practice after implementation of the guideline of expectant management. For examples, Duvekot et al. [15] showed that median prolongation of pregnancy was 2 days (interquartile range 1–3 days) in the group of expectant management of severe preeclampsia between 28 and 34 weeks. Bombrys et al. [9] reported that median for days of prolongation was 5 days (range 3 to 35). A very recent large study conducted by Sanjanwala et al. [16] compared pregnancy outcomes in women with severe preeclampsia before and after implementation of the ACOG hypertensive guidelines. They showed that the median length of maternal hospitalization was different in only one day (6 vs. 7 days). In brief, in the service setting, like our study, expectant management of preeclampsia with severe features can prolong pregnancy duration in approximately 2–7 days, varying among studies. Hypothetically, the shorter prolongation than that seen in the research settings may be associated with the lower threshold of making decisions on stopping expectant management due to higher safety awareness or increased fear of maternal and fetal complications in actual practice and during clinical changes, especially in low-resource settings.

Based on a systematic review [2,17], the benefit of expectant management is still unclear. Though expectant management resulted in pregnancy prolongation of approximately 7–15 days, perinatal mortality rate was comparable in some studies [4,7,9,16], including a large RCT reported by Vigil-De Gracia et al. [7] in spite of 10-day prolongation, and slightly decreased in some studies [1,5,13,17]. Likewise, maternal morbidity was comparable in most studies [1,4,13,14] and increased in some studies [5,7,9]. In summary of the systematic review, expectant management of preeclampsia with severe features between 24 and 34 weeks of gestation may be associated with decreased perinatal morbidity. However, this evidence was based on limited data from relatively low-quality trials. Further large, high-quality trials are needed to confirm or refute these findings and establish whether this approach is safe for the mother both in research settings and service settings in actual practice.

Effectiveness of expectant management demonstrated in this study was comparable with that in the previous studies based on actual practice but seemed to be inferior when compared to most prospective control trials. It may be concluded that the effectiveness of expectant management may not be reproducible in actual practice, in spite of the same management protocol, using the same criteria for expectant management.

In brief, our study suggests that, due to only three days of prolongation achieved by expectant management, this approach adds only minimal to aggressive management by induction of labor after a complete course of corticosteroids. From this point of view, expectant management of preeclampsia with severe features in low-resource settings should be strongly considered to weigh maternal risks. It should be emphasized that the outcomes of this study were based on actual practice, different from most previous studies that were conducted in the milieu of research settings with high standards of care. Accordingly, based on our study, the benefits of expectant management quoted in several studies might not be reproducible in actual practice, as seen in our study. No reproducibility was noted in the study comparing the outcomes before and after adopting guidelines for expectant management of severe preeclampsia at the University of Alabama at Birmingham, which showed that perinatal outcomes were similar before and after implementation of the guidelines [16].

The new insight gained from this study is that (1) UPCI is an independent factor that is significantly associated with expectant management failure. In practice, UPCI is more practical than 24 h urine protein, especially in situations where the choice of treatment must be made very urgently. UPCI may be one of the factors that helps to identify cases that are suitable for expectant management or the decision of referral for expectant management where MFM specialists are available. (2) Expectant management in the service setting or actual implementation might be less effective than that based on research settings with highly intensive care. Additionally, the benefit and risk of expectant management in actual practice of service settings in terms of maternal and fetal morbidity is still unclear.

The strengths of this study include the following: (1) Although the outcomes in this study are not as good as in previous studies, the results represent real practice of expectant management of preeclampsia with severe features in service settings instead of research settings, as used in many published studies. (2) Detailed information was obtained from a comprehensive review of the medical records and not just from the crude obstetric database. However, this study also has limitations, which are as follows: (1) Because of its retrospective nature, some essential data were missing in some cases. (2) The sample size was too small to show a significant small difference in some rare outcomes, if existing, such as perinatal death, maternal death, or eclampsia.

Research implications: The studies on this topic may be divided into two categories: the studies based on research settings (including prospective studies or RCTs) and the studies based on the service setting or actual practice. The effectiveness of management using these two entities seems to be different. Though this study seems to have negative results, it reflects the outcomes of actual practice. However, the studies on actual practice are still required in the literature. Accordingly, this could be a resource for future meta-analysis of the studies based on actual practice, which better represents the effectiveness than the studies based on research settings.

## 5. Conclusions

The rate of pregnancy prolongation for at least 48 h with expectant management was 65%, with a median prolongation time of 2.9 days, and 88% had no maternal complications. A new insight gained from this study is that high UPCI at admission is an independent factor for prolongation of less than 48 h. This study shows that the duration of prolongation of 7–14 days of conservative management in most previous studies in research settings may not be reproducible in actual practice of service settings. Nevertheless, three days of prolongation is relatively safe for the mother and might benefit the fetuses from corticosteroid administration for lung maturity. Probably because of short duration of prolongation, both maternal and neonatal outcomes were not significantly different between the group of failed and successful conservative management, though the neonatal outcomes seem to be better in the group of success in prolongation of more than 48 h. Importantly, the benefit and risk of expectant management in actual practice of service settings in terms of maternal and fetal morbidity are still unclear, and further studies are needed.

## Figures and Tables

**Table 1:** Baseline demographic data of the group of prolongation of less than 48 h and that of at least 48 h (* Mann–Whitney U test, # chi-square test).

Parameter Median (Range), IQR	Prolongation < 48 h (N = 35)	Prolongation ≥ 48 h (N = 65)	*p*-Value
Maternal age	31 (24–46), 9	32 (21–45), 11	0.89 *
GA at admission	30 (26–33), 4	29 (23–34), 5	0.25 *
SBP at admission	170 (154–202), 29	170 (137–223), 18	0.28 *
DBP at admission	110 (96–130), 20	109 (91–141), 11	0.35 *
UPCI at admission (N 64)	4.52 (0.24–12), 6.7	1.07 (0.12–19), 3.62	0.006 *
Maternal with underlying disease (%)	13 cases, 37.1%	23 cases, 35.4%	0.86 #

**Table 2:** Pregnancy outcomes of the group of prolongation of less than 48 h and that of at least 48 h (* Student’s T test, # chi-square test).

Outcomes	Prolongation < 48 h (N = 35)	Prolongation ≥ 48 h (N = 65)	*p*-Value
Mean gestational age (week) at delivery (mean ± SD)	29.8 ± 2.2	30.3 ± 2.6	0.334 *
Birth weight (g); (mean ± SD)	1092 ± 392	1234 ± 482	0.152 *
Route of delivery (n; %)	-		0.312 #
Normal vaginal delivery	4 (11.4%)	15 (23.1%)	
Vacuum extraction	1 (2.9%)	3 (4.6%)	
Cesarean delivery	30 (85.7%)	47 (72.3%)	
Maternal complications (n; %)	5 cases, 14.3%	6 cases, 9.2%	0.380 #
Postpartum hemorrhage	1	4	
Pulmonary edema	1	1	
HELLP	0	1	
PRES	1	0	
Magnesium toxicity	1	0	
Multiple	1	0	
Low Apgar score at 1 min (<7) (n; %)	29 (82.9%)	38 (58.5%)	0.013 #
Low Apgar score at 5 min (<7) (n; %)	16 (45.7%)	18 (27.7%)	0.070 #
Neonatal complications (n; %)	25 (71.4%)	40 (61.5%)	0.189 #
RDS	17	28	
Growth restriction	1	0	
Birth injury	0	1	
RDS + growth restriction	3	7	
RDS + NEC	1	0	
RDS + IVH	0	1	
RDS + BPD +growth restriction	1	0	
RDS + pulmonary hemorrhage	1	1	
RDS + birth injury	0	1	
RDS + birth asphyxia	1	1	
Perinatal death	1	1	

BPD: bronchopulmonary dysplasia; HELLP: hemolysis, elevated liver enzymes, and low-platelets syndrome; IVH: intraventricular hemorrhage; NEC: necrotizing enterocolitis; PRES: posterior reversible encephalopathy syndrome; RDS: respiratory distress syndrome.

**Table 3:** Univariate and multivariate analysis of factors that can predict pregnancy prolongation for at least 48 h with expectant treatment.

Parameter	Univariate Analysis			Multivariate Analysis		
	*p*-Value	Odds Ratio	95% CI	*p*-Value	Odds Ratio	95% CI
Maternal age	0.98	1.001	(0.94–1.07)	0.33	0.96	(0.87–1.05)
GA at admission	0.22	0.9	(0.76–1.07)	0.22	0.88	(0.71–1.08)
Maternal with any underlying disease	0.86	0.93	(0.4–2.18)	0.84	0.89	(0.29–2.72)
SBP at admission	0.15	0.98	(0.95–1.01)	0.98	1.00	(0.95–1.05)
DBP at admission	0.31	0.98	(0.94–1.02)	0.65	0.98	(0.91–1.06)
UPCI at admission	0.04	0.87	(0.77–0.99)	0.04	0.86	(0.75–0.99)

## Data Availability

The datasets analyzed in the current study are available from the corresponding author upon reasonable request.
